# Clinical Relevance of HLA Gene Variants in HBV Infection

**DOI:** 10.1155/2016/9069375

**Published:** 2016-05-08

**Authors:** Li Wang, Zhi-Qiang Zou, Kai Wang

**Affiliations:** ^1^Infectious Disease Hospital of Yantai, 62 Huanshan Road, Zhifu District, Yantai, Shandong 264001, China; ^2^Hepatology Department, Qilu Hospital of Shandong University, 44 Wenhua West Road, Lixia District, Jinan, Shandong 250012, China

## Abstract

Host gene variants may influence the natural history of hepatitis B virus (HBV) infection. The human leukocyte antigen (HLA) system, the major histocompatibility complex (MHC) in humans, is one of the most important host factors that are correlated with the clinical course of HBV infection. Genome-wide association studies (GWASs) have shown that single nucleotide polymorphisms (SNPs) near certain HLA gene loci are strongly associated with not only persistent HBV infection but also spontaneous HBV clearance and seroconversion, disease progression, and the development of liver cirrhosis and HBV-related hepatocellular carcinoma (HCC) in chronic hepatitis B (CHB). These variations also influence the efficacy of interferon (IFN) and nucleot(s)ide analogue (NA) treatment and response to HBV vaccines. Meanwhile, discrepant conclusions were reached with different patient cohorts. It is therefore essential to identify the associations of specific HLA allele variants with disease progression and viral clearance in chronic HBV infection among different ethnic populations. A better understanding of HLA polymorphism relevance in HBV infection outcome would enable us to elucidate the roles of HLA SNPs in the pathogenesis and clearance of HBV in different areas and ethnic groups, to improve strategies for the prevention and treatment of chronic HBV infection.

## 1. Introduction

Certain host gene variants, including human leukocyte antigen (HLA) classes I and II alleles as well as non-HLA genes, influence the natural history of hepatitis B virus (HBV) infection. The HLA system, the major histocompatibility complex (MHC) in humans, is one of the most important host factors correlated with the clinical course of HBV infection. The highly polymorphic HLA classes I and II genes can affect the ability of HLA molecules to trigger immune responses, which affects the outcome of infection by any given pathogen [1]. HBV peptides presented by HLA class I molecules to cytotoxic T lymphocytes (CTLs) are critical in the eradication of HBV infection by boosting CTL ability to identify and kill HBV-infected cells [2, 3].

The discordance between HBV-specific CD8^+^ T cell repertoires present in different ethnic groups, such as Caucasian and Chinese subjects, was shown to reflect the ability of HLA micropolymorphisms to diversify T cell responses [4]. Previous studies have revealed the associations of certain HLA class I genes with the course of HBV infection [5].

HLA class II molecules are cell surface glycoproteins of antigen presenting cells (APCs), which are responsible for presenting exogenous antigens to CD4^+^ T-helper cells. Antigen presentation efficiency may vary due to gene loci [6]. Genome-wide association studies (GWASs) have shown that single nucleotide polymorphisms (SNPs) near HLA-DP, HLA-DQ, and HLA-DR loci are significantly correlated with HBV infection outcomes [7–9]. HLA class II gene variations are strongly associated with not only persistent HBV infection but also spontaneous HBV clearance and seroconversion [10, 11], disease progression, and the development of liver cirrhosis (LC) and HBV-related hepatocellular carcinoma (HCC) in chronic hepatitis B (CHB) [7, 12, 13]. Such variations also affect interferon (IFN) and nucleot(s)ide analogue (NA) treatment efficacy and the response to HBV vaccines. Meanwhile, discrepant conclusions are reached in different cohorts [14]. For instance, the candidate variant rs9277535 (550A/G) in the 3′ untranslated region (3′UTR) of the HLA-DPB1 gene, associated most significantly with CHB and HBV infection outcomes in Asians, was shown to have a minimal effect on HBV recovery in European American and African American subjects [15]. It is therefore critical to identify the associations of specific HLA allele variants with disease progression and viral clearance in chronic HBV infections among different ethnic groups.

A deeper understanding of HLA polymorphism relevance in HBV infection outcome would help elucidate the role of HLA SNPs in the pathogenesis and clearance of HBV in different areas and ethnicities, to improve strategies for the prevention and treatment of chronic HBV infection. This review summarizes the reported associations of HLA polymorphisms with susceptibility to HBV infection, resolution, disease progression, and antivirus treatment efficacy and response to HBV vaccines.

## 2. Associations of HLA Gene Variants with Susceptibility and Persistence of HBV Infection

Though several GWASs have revealed the association of gene variants in the HLA region with chronic HBV infection, the susceptibility gene loci and potential mechanisms have not been fully identified. A comparative review showed that HLA-DRB1 ^*∗*^11/ ^*∗*^12 alleles and DQB1 ^*∗*^0301 are associated with HBV persistence globally [14]. A Chinese study by Zhu et al. [16] identified four loci that independently drive chronic HBV infection, including HLA-DP*β*1 positions 84–87, HLA-DR*β*1 ^*∗*^13 sites 71 and rs400488, and HLA-C position 15. In another study [17], after genotyping 140 SNPs within the HLA-DP/DQ genomic region in a total of 1657 HBV-positive patients and 1456 HBV-negative controls, 76 SNPs and 5 LD blocks were identified in HLA-DP/DQ clusters, independent of each other, which are significantly associated with HBV infection; rs9277535 in HLA-DPB1 was found to be the most significant locus. Chang et al. [18] found that rs9277535 (HLA-DPB1), rs9276370 (HLA-DQA2), rs7756516 and rs7453920 (HLA-DQB2), and rs9366816 near HLA-DPA3 are significantly associated with persistent HBV infection, especially the “T-T” haplotype composed of rs7756516 and rs9276370 that is more prevalent in severe disease subgroups and associated with nonsustained therapeutic response (*P*  =  0.0262) in male Taiwan Han Chinese individuals. DQB1 ^*∗*^0301 and DQB1 ^*∗*^0303 are correlated with continuous infection in Xinjiang Uygur Chinese subjects [8].

A transethnic association analysis [19] performed among Asian populations, including Japanese, Korean, Hong Kong, and Thai subjects, revealed Asian-specific associations of HLA-DPA1 and HLA-DPB1 alleles/haplotypes with HBV infection and disease progression. The latter study identified a new risk allele HLA-DPB1 ^*∗*^09:01 and a new protective allele DPB1 ^*∗*^02:01 in chronic HBV infection. An American study [15] showed that the HLA-DPB1 3′UTR 496GG genotype confers latent susceptibility to persistent HBV infection and is also associated with significantly higher levels of HLA-DP surface protein expression in healthy donors, suggesting that differences in HLA-DP expression may increase the risk for persistent HBV infection in European Americans and African Americans. A prospective study [20] showed that class II alleles DQA1 ^*∗*^0501 (*P* = 0.05) and DQB1 ^*∗*^0301 (*P* = 0.01), the two-locus haplotype consisting of the latter two alleles, and the three-locus haplotype DQA1 ^*∗*^0501, DQB1 ^*∗*^0301, and DRB1 ^*∗*^1102 (OR = 10.7; *P* = 0.01) are significantly associated with persistent HBV infection in an African American cohort.

In addition, HBV persistence was shown to be associated with class II allelic homozygosity. Interestingly, three SNPs belonging to the HLA-DQ region (rs2856718, rs7453920, and rs9275572), shown to display increased susceptibility to chronic HBV infection, were detected in Saudi Arabian patients [21]. Meanwhile, DRB1 ^*∗*^08 and DRB1 ^*∗*^09 alleles, which are susceptible to HBV infection, were found in Brazilian populations determined in young and male blood donors [9]. A study [22] identified 2 risk alleles in MHC loci, namely, HLA-DPA1 (rs3077) and HLA-DPB1 (rs9277535), using salivary DNA extracted with a modified protocol from blood samples in Chinese patients. This provides a new noninvasive screening method for identifying risk loci.

Furthermore, HLA gene variants are also associated with susceptibility to vertical transmission. Multiple factors, including HBV structure and DNA level, placental barrier, the immune status of the mother, and the genetic background of the newborn infant, determine the susceptibility to intrauterine HBV infection. Xu et al. [23] assessed 15 HLA-DR alleles and found HLA-DRB1 ^*∗*^07 to be the only one associated with infant susceptibility to intrauterine HBV infection.

On the other hand, gene polymorphisms of some HLA loci confer protective effects against persistent HBV infection. It was shown that the HLA-DPA1 and HLA-DPB1 genes are significantly associated with protective effects against CHB in Japanese, Korean, and other Asian populations, including Chinese and Thai individuals [19]. Cross-sectional studies showed that HLA-DRB1 ^*∗*^1301-02 are associated with protection against persistent HBV infection in Gambia, Germany, and Korea [24–26]. China has the highest HBV prevalence rate in the world, with different ethnic populations. Wang et al. [27] demonstrated that, in two independent case-control studies, HLA-DP A alleles of both rs3077 and rs9277535 significantly decrease the risk for CHB in Chinese Han subjects, while HLA-DP rs9277535 is associated with decreased risk in Chinese Zhuang subjects. In addition, HLA-DQB1 ^*∗*^0201 is HBV resistance gene in Xinjiang Uygur ethnic groups of China [8]. Although the most significant associations were observed for HLA-DPA1 rs3077 and HLA-DPB1 rs9277535 A alleles (decreased risk for HBV infection in Asian populations), only a highly significant association of HLA-DPA1 rs3077 with HBV infection was observed in Caucasians [28].

## 3. Associations of HLA Gene Variants with Spontaneous HBsAg Clearance and HBV Eradication

Spontaneous HBsAg seroclearance occurs in a very small proportion of patients with chronic HBV infection. The mechanisms of spontaneous HBV clearance are determined by the interactions between HBV and the host immune response, including innate and adaptive immune responses, which are affected by specific HLA gene polymorphisms that alter peptide epitope binding. For instance, HLA-DPA1 and HLA-DPB1, which encode the HLA-DP *α* and *β* chains, may be involved in antigen presentation to CD4^+^ positive T lymphocytes, which is important for HBV clearance [27, 29]. HLA-DR13 is consistently associated with HBV clearance globally [14, 24, 30, 31]. A meta-analysis indicated that subjects harboring at least one A allele of HLA-DPB1 rs9277535 and HLA-DPA1 rs3077 variants have increased susceptibility to spontaneous HBV clearance compared with those with G alleles [7]. Three SNPs of the HLA-DP gene, including rs9277535, rs7453920, and rs2856718, confer increased frequency of HBsAg clearance in China [10]. Hu et al. [12] showed a significantly higher proportion of the rs9277534 minor allele A in spontaneous HBV clearance than in the HBV persistent infection group (*P* < 0.0001). Genotypic analyses [5] showed that GA and AA genotypes are associated with spontaneous HBV clearance. In addition, HLA-B ^*∗*^13:01:01G frequency is associated with spontaneous HBsAg clearance in a Qidong Han Chinese population. HBV carriers with rs9277535 non-G/G genotype and GA haplotype have a higher chance to clear HBsAg in Chinese subjects of Taiwan [32]. The rs3077 and rs9277542 alleles in the HLA-DPA1 in HLA-DPB1 genes, respectively, confer protective effects on HBV infection and clearance in Japanese and Korean populations [19]. In a study assessing European and African American populations, rs9277534 rather than rs9277535 in the HLA-DPB1 3′UTR was shown to be associated with HBV recovery [33]. A meta-analysis of 62,050 subjects from 29 case-control studies showed that rs3077 and rs9277535 in HLA-DP significantly decrease HBV infection risks and increase HBV clearance [34]. The 496A/G variant has a stronger effect than any individual HLA-DPB1 or DPA1 allele, as well as other HLA alleles associated with HBV recovery in European American cohort [15]. Li et al. [35] showed that HLA-B ^*∗*^15 and DRB1 ^*∗*^11 and DRB1 ^*∗*^14 are associated with spontaneous recovery in patients with HBV subgenotype C2 infection in Northeast China.

HLA-DR2, HLA-DR ^*∗*^0406, HLA-B ^*∗*^4001, and HLA-DR7 antigens are associated with protective effect on acute HBV infection [19, 36, 37]. It is known that patients who successfully resolve acute hepatitis B infection develop strong HLA classes I and II restricted T cell response, which is weak or absent in patients with chronic hepatitis B [38, 39]. In Spain, Cotrina et al. [40] found that the HLA-DRB1 ^*∗*^1301 and DRB1 ^*∗*^1302 alleles are associated with infection resolution in acute hepatitis B. Li et al. [35] also showed that HLA-B ^*∗*^07 and DRB1 ^*∗*^13 may protect subjects from HBV infection. HLA-DQ rs2856718G and rs9275572A are strongly associated with decreased risk of chronic HBV infection and natural clearance [41].

## 4. Associations of HLA Gene Variants with Early Hepatitis B e Antigen (HBeAg) Seroconversion

HBeAg seroconversion mainly depends on patient age at infection and the host immune responses. It was shown that the functional stage of dendritic cells (DCs) plays an important role in HBeAg seroconversion [42]. DCs are the most effective antigen presenting cells and play a pivotal role in antiviral response induction. Exogenous antigens are phagocytized and then loaded on both HLA classes I and II by DCs. Therefore, HLA gene variants may influence host induced early HBeAg seroconversion. A long-term cohort study demonstrated that HLA class I antigen B61 and class II antigen DQB1 ^*∗*^0503 are associated with early HBeAg seroconversion in CHB children in Taiwan [43]. Although the HLA-DPA1 SNP did not show a statistically significant association with early HBeAg seroconversion in Japanese children, it tended to increase the likelihood of achieving early spontaneous HBeAg seroconversion [44]. In an African HIV-positive cohort, it was suggested that HLA-A alleles alone, other than HLA-B or HLA-C, indeed predict HBeAg status (AUC = 0.73, *P* = 0.002) [45]. These results emphasize the role of the CD8^+^ T cell response in HBV control.

## 5. Association of HLA Gene Variants with the Risk for Developing Liver Cirrhosis and HBV-Related Hepatocellular Carcinoma

HLA gene variations are strongly associated with not only HBV infection persistence or clearance but also disease progression and the development of liver cirrhosis (LC) and HBV-related hepatocellular carcinoma (HCC). HLA-DQ polymorphism analysis [41] using matrix-assisted laser desorption/ionization time of flight mass spectrometry showed that rs9275572A is associated with the development of cirrhosis and HCC (OR = 0.632,* P* = 0.008). Of the SNPs reported in HBV-related HCC GWASs, rs9267673 near C2, rs2647073 and rs3997872 near HLA-DRB1, and rs9275319 near HLA-DQ were found to be significantly associated with the risk for HBV-related LC [46], suggesting that gene variants associated with HBV-related hepatocarcinogenesis may already play an important role in the progression from CHB to LC. Therefore, understanding HLA genetic background would help improve current HCC surveillance programs in HBV-infected patients.

GWAS on genetic susceptibility of HBV-related HCC indicated two consistently identified tagging SNPs around HLA-DQ/DR [47, 48]. A multicenter case-control study including 1,507 HBV-related HCC cases and 1,560 HBV persistent carriers as controls showed that HBV carriers infected with HBV genotype C and carrying HLA-DQ/DR SNPs (rs9272105 AA genotype, rs9275319 AA genotype) have a relatively high risk for HCC [49]. Other studies [10, 13] suggested that HLA-DP rs3077 and rs9277535 polymorphisms are associated with HCC susceptibility in Asian individuals. Four SNPs (rs17875380, rs41557518, rs114465251, and rs115492845), in nonclassical class I alleles, were shown to be associated with altered susceptibility to HBV or HCC, while HLA-F ^*∗*^01:04, HLA-G ^*∗*^01:05N, and HLA-E ^*∗*^01:01 are associated with hepatitis B or hepatitis B complicated with HCC. Six of 16 designated HLA-E, HLA-G, and HLA-F haplotypes were shown to be associated with risk for hepatitis B or HCC [50].

On the other hand, HLA gene variations also decrease the risk for cirrhosis and HCC. Doganay et al. [51] found in a multivariate logistic regression analysis that DRB1 ^*∗*^07 is a significant negative predictor of cirrhosis (*P* = 0.015). This may be due to the fact that a polymorphic amino acid sequence in DRB1 ^*∗*^07 alters interaction with the T cell recognition site. Mohamadkhani et al. [29] revealed that the rs2856718 variant significantly diminishes the host risk for HCC. Zhang et al. [13] also indicated that the HLA-DP SNP rs3077 might act beneficially against HCC susceptibility. In another study, HLA-DP rs3077, rs9277535, and rs7453920 also showed no association with HCC development [52].

Killer cell immunoglobulin-like receptors (KIRs) are involved in the regulation of NK cell activation through recognition of specific HLA class I allotypes. In a multivariate Cox model, Pan et al. [53, 54] suggested that KIR and HLA genetic background can influence the age at HCC onset in male patients and is associated with HCC incidence in patients with HBV infection.

## 6. HLA Gene Variants Associated with Efficacy of Interferon- (IFN-) ***α*** and NAs Treatment

Interferon- (IFN-) *α* is the first-line therapy for CHB patients but initiates a complete response only in a minority of patients. HLA gene variants have also been shown to be associated with response to IFN-*α* treatment in CHB patients. Different haplotypes of the same SNP may be associated with different clinical treatment outcomes. It was shown that the “G-C” haplotype of the five SNPs, including rs9277535 (HLA-DPB1), rs9276370 (HLA-DQA2), rs7756516 and rs7453920 (HLA-DQB2), and rs9366816 near HLA-DPA3, is associated with sustained therapeutic response to IFN-*α* treatment in male Han Taiwanese subjects (*P*  =  0.0132; OR * *= * *2.49) [18]. In a large cohort of Caucasian chronic hepatitis B patients infected with the HBV genotype A or D, Brouwer et al. demonstrated that HLA-DPB1 polymorphisms are independently associated with both virological and serological responses to PEG-IFN therapy at 6 months after treatment.

HLA-DPA1 and HLA-DPB1 haplotype block GG showed comparable results for virological and combined response [55]. Han et al. [56] suggested that HLA-DRB1 ^*∗*^14 allele may be associated with a high rate of the response of CHB patients to IFN treatment. Compared with other HLA-DQB1 alleles, HLA-DQB1 ^*∗*^07 may be associated with low response rate to IFN.

Cheng et al. [57] showed by multivariate analysis that, at 6 months of PEG-IFN-*α* therapy and 6 months after therapy, rs3077-GG genotype is independently associated with higher HBeAg loss and anti-HBeAb seroconversion rates; meanwhile, the rs9277535-GG genotype was independently associated with declined HBV DNA levels in Chinese patients with CHB. Similar results were observed in Taiwan [58].

The HLA-DQ locus rs9275572 is a predictor of viral and biochemical responses to lamivudine (LAM) therapy in Han Chinese subjects [41]. Hosaka et al. [59] demonstrated an association of HLA-DP polymorphisms with ≥2 A alleles at rs3077 and rs9277535 and decreased HBsAg levels and seroclearance among HBeAg-positive Japanese CHB patients treated with LAM. Meanwhile, the HLA-DRB1 ^*∗*^010101 allele is closely associated with poor virological response to initial LAM therapy in Korean CHB patients [60]. Chang et al. [18] also showed that, in patients with the TT genotype of rs9276370 (HLA-DQA2), there is a higher nonsustained response rate, especially in the LAM (*P* = 0.0074) and PEG-IFN-*α*-2a (*P* = 0.0814) groups, rather than in entecavir treated individuals. A randomized clinical trial [61] assessing PEG-IFN-*α*-2b with or without entecavir in patients with HBeAg-negative CHB revealed the GG genotype of rs3077 (HLA-DPA1) as an independent predictor of therapeutic response.

## 7. HLA Gene Variants Associated with Response to Hepatitis B Vaccination

Accumulating evidence shows that certain HLA types are associated with decreased or increased antibody response to hepatitis B vaccines in different individuals. A meta-analysis [62], including 774 potentially relevant articles and a total of 2308 subjects (1215 responders, 873 nonresponders, and 220 control populations) and assessing the effect of HLA on immunological response to hepatitis B vaccines in healthy individuals, showed that, for DRB1 alleles, the three HLA variants DRB1 ^*∗*^01, DRB1 ^*∗*^1301, and DRB1 ^*∗*^15 are associated with significantly increased antibody response to hepatitis B vaccines, with pooled ORs of 2.73, 5.94, and 2.29, respectively. Meanwhile, DRB1 ^*∗*^03 (DRB1 ^*∗*^0301), DRB1 ^*∗*^04, DRB1 ^*∗*^07, and DRB1 ^*∗*^1302 showed opposite results. For DQB1 alleles, DQB1 ^*∗*^05 (DQB1 ^*∗*^0501), DQB1 ^*∗*^06, and DQB1 ^*∗*^0602 were shown to be associated with markedly increased antibody response to hepatitis B vaccine, with pooled ORs of 1.85, 2.35, 2.34, and 3.32, respectively; DQB1 ^*∗*^02 (pooled OR = 0.27) showed opposite results. Mert et al. [63] found positive correlations between four HLA-DR (HLA-DRB1 ^*∗*^04X, DRB1 ^*∗*^0401X, DRB1 ^*∗*^11/13, and DRB1 ^*∗*^0401X0201) haplotypes and nonresponders but a negative correlation with one class I (HLA-B13) in Turkey. In Korean infants [64] who received HBV vaccination, HLA-A ^*∗*^33, B62, DRB1 ^*∗*^04, and DRB1 ^*∗*^07 alleles showed positive associations with nonresponsiveness (<10 mIU/mL) or low antibody titers (<100 mIU/mL), while alleles A ^*∗*^02 and DRB1 ^*∗*^08 showed negative associations. After stratification by other associated alleles at different loci, B62 and DRB1 ^*∗*^07 were still independently associated with nonresponsiveness. So, upon evaluating the response to HBV vaccination, different HLA types of ethnic groups should be taken into consideration; HLA gene frequencies of distinct ethnic groups should be examined in further large-scale population-based studies [65].

## 8. Summary and Perspectives

Overall, the complicated nature history of HBV infection makes it necessary to find clinical and genetic markers to help predict individuals at higher risk to develop CHB and worse outcomes such as LC and HCC. The HLA system is an integral component of the host immune response. The highly polymorphic HLA genes are key factors in the activation of the immune response against HBV infection through their enormous capacity of attracting and binding viral peptides. HLA gene variations are associated not only with susceptibility or resistance to HBV infection but also with spontaneous HBV clearance, disease progression, efficacy of antiviral treatment, and response to HBV vaccines. Furthermore, specific HLA allele variants may have different impact on clinical outcomes of chronic HBV infections among different ethnic subjects. Identifying the associations of specific HLA allele variants with disease progression or viral clearance in chronic HBV infections among different ethnic populations needs further assessment in larger scale controlled clinical trials.

Finally, upon evaluating the impact of HLA gene variants on HBV infection, SNP-SNP interactions between HLA and other host genes such as granulysin (GNLY) SNPs [66] and polymorphisms in toll-like receptor-interferon (TLR-IFN) [67] pathway genes and HBV mutations [68] should also be kept in mind.
